# Supplementary material for: Dynamic transcriptomic profiles of zebrafish gills in response to zinc supplementation
Source: BMC Genomics. 2010 Oct 11;11:553. doi: 10.1186/1471-2164-11-553 (PMC3091702; doi:10.1186/1471-2164-11-553)
Supplement: Additional file 2 — Interactive Direct Interaction Network representing the molecular interactions between zinc, copper, iron, calcium and proteins encoded by transcripts changed by zinc supplementation. Mini web-site containing index.html and hyperlinked pages in subdirectory describing a Direct Interaction Network automatically generated based on curated interactions contained within the proprietary PathwayArchitect database. Ovals represent proteins and the circles symbolize metal ions. Objects are coloured by their abundance in zebrafish at the time-point they were significantly different from the control is a scale from -4 fold (dark green) to +4 fold (dark red). Where significant differences were found at more than one time-point, the colour overlay shows expression at the first instance. Dark blue squares denote 'binding', and light blue squares 'expression'; green squares stand for 'regulation', green diamonds for 'metabolism', and green circles for 'promoter binding'. Arrow heads indicate directionality of the interaction where annotated. All nodes and edges can be further interrogated by selecting the relative area of the image. [file 1471-2164-11-553-S2.zip › PathwayArchitect Zn xs DIN/394822.html]

# BINDING:

|  |  |
| --- | --- |
| Type | BINDING |
| Effect | None |


---

|  |  |
| --- | --- |
| Score | 0 |


---

|  |  |
| --- | --- |
| Reference Count | 14 |


---

|  |  |
| --- | --- |
| Mechanism | Unknown |


---

|  |  |
| --- | --- |
| Reference:0 || Sentence | "In contrast with the results for serum iron, the levels of ferritin, total iron binding capacity (transferrin), zinc, copper and coeruloplasmin were not altered in any subject during the insulin infusion or at 24 h following discontinuation of the infusion." |
| PMID | 3548194 |
| Year | 1987 |
| Species | Human |
| Journal | Acta Endocrinol (Copenh) |
| RefScore | 1 |
| Source | PArchNLP |
  |
|  |  |


---

|  |  |
| --- | --- |
 Reference:1 || Sentence | "Copper protected rats against the action of lead on the following hematologic parameters: hemoglobin concentration, hematocrit, iron concentration, total and latent iron binding capacity, transferrin saturation index, copper concentration and ceruloplasmin activity." |
| PMID | 2616386 |
| Year | 1989 |
| Species | Rat |
| Journal | Pol J Pharmacol Pharm |
| RefScore | 2 |
| Source | PArchNLP |
  ||  |  |


---

|  |  |
| --- | --- |
 Reference:2 || Sentence | "Major recent developments include the synthesis of a number of unusual and biologically relevant complexes of copper and iron using elaborate N-donor and O-donor ligands." |
| PMID | 11282346 |
| Year | 2001 |
| Journal | Curr Opin Chem Biol |
| RefScore | 1 |
| Source | PArchNLP |
  ||  |  |


---

|  |  |
| --- | --- |
 Reference:3 || Sentence | "No significant changes were observed in bilirubin, hemoglobin, ceruloplasmin, transferrin, copper, or total iron binding capacity." |
| PMID | 454320 |
| Year | 1979 |
| Species | Human |
| Journal | Aviat Space Environ Med |
| RefScore | 0 |
| Source | PArchNLP |
  ||  |  |


---

|  |  |
| --- | --- |
 Reference:4 || Sentence | "No significant differences were found in serum iron, total iron binding capacity, marrow iron, plasma copper or hemoglobin type between affected and normal calves." |
| PMID | 2412677 |
| Year | 1985 |
| Journal | Can J Comp Med |
| RefScore | 0 |
| Source | PArchNLP |
  ||  |  |


---

|  |  |
| --- | --- |
 Reference:5 || Sentence | "Biochemical parameters included serum albumin, total iron-binding capacity, copper, zinc, and creatinine height index." |
| PMID | 3934976 |
| Year | 1985 |
| Species | Human |
| Journal | Am J Obstet Gynecol |
| RefScore | 0 |
| Source | PArchNLP |
  ||  |  |


---

|  |  |
| --- | --- |
 Reference:6 || Sentence | "Kinetic analyses further suggest that albumin competes with substrate (ferrous iron) for binding to the functional copper of the serum ferroxidases." |
| PMID | 6441732 |
| Year | 1984 |
| Species | Human |
| Journal | Int J Biochem |
| RefScore | 1 |
| Source | PArchNLP |
  ||  |  |


---

|  |  |
| --- | --- |
 Reference:7 || Sentence | "BACKGROUND: Abnormal interactions of copper or iron in the brain with metal-binding proteins (such as amyloid-beta peptide [Abeta] or neuromelanin) that lead to oxidative stress have emerged as important potential mechanisms in brain ageing and neurodegenerative disorders." |
| PMID | 15207800 |
| Year | 2004 |
| Species | Human |
| Journal | Lancet Neurol |
| RefScore | 1 |
| Source | PArchNLP |
  ||  |  |


---

|  |  |
| --- | --- |
 Reference:8 || Sentence | "We then briefly consider iron and copper metabolism in mammals, before looking at iron-copper interactions in mammals, particularly man, and conclude with the reflection that, as in Greek and Roman mythology, a better understanding of the potentially positive interactions between Mars (iron) and Venus (copper) can only be to the advantage of our species." |
| PMID | 11508852 |
| Year | 2001 |
| Species | Human |
| Journal | Biometals |
| RefScore | 1 |
| Source | PArchNLP |
  ||  |  |


---

|  |  |
| --- | --- |
 Reference:9 || Sentence | "Manganese, iron, cobalt, nickel, copper and zinc complexes of isatin-beta-thiosemicarbazone (H2L) have been synthesized and spectroscopically characterized The X-ray crystal structures of two nickel complexes, namely [Ni(HL)2]." |
| PMID | 10212992 |
| Year | 1999 |
| Species | Human |
| Journal | J Inorg Biochem |
| RefScore | 0 |
| Source | PArchNLP |
  ||  |  |


---

|  |  |
| --- | --- |
 Reference:10 || Sentence | "Other studies included analysis of serum iron, total iron-binding capacity, ferritin, copper, vitamins B6 and B12, and folate levels." |
| PMID | 10524457 |
| Year | 1999 |
| Species | Human |
| Journal | J Pediatr Hematol Oncol |
| RefScore | 0 |
| Source | PArchNLP |
  ||  |  |


---

|  |  |
| --- | --- |
 Reference:11 || Sentence | "Duodenal uptake and transfer of 59Fe and 65Zn and absorptive interactions between iron, zinc, cobalt, and copper were studied in sla mice and in genetically normal Swiss albino control mice." |
| PMID | 6498623 |
| Year | 1984 |
| Species | Mouse |
| Journal | Can J Physiol Pharmacol |
| RefScore | 0 |
| Source | PArchNLP |
  ||  |  |


---

|  |  |
| --- | --- |
 Reference:12 || Sentence | "CBC, serum albumin, serum total protein, serum iron, iron binding capacity, serum zinc and copper, and serum vitamin C were studied weekly for 4 weeks and biweekly for 8 weeks." |
| PMID | 1634717 |
| Year | 1992 |
| Species | Human |
| Journal | J Am Geriatr Soc |
| RefScore | 0 |
| Source | PArchNLP |
  ||  |  |


---

|  |  |
| --- | --- |
 Reference:13 || Sentence | The results reveal that sulfuric acid was an effective extractant and that the copper fraction in the extracted sludge shifted from being mostly bound to the Fe-Mn oxides and organic matter, to being mostly bound to organic matter and remaining as a residue during acid extraction. |
| PMID | 15811688 |
| Year | 2005 |
| Journal | J Hazard Mater |
| RefScore | 1 |
| Source | PArchNLP |
  |


---

|  |  |
| --- | --- |
